# Disseminated tuberculosis in a patient treated with a JAK2 selective inhibitor: a case report

**DOI:** 10.1186/1756-0500-5-552

**Published:** 2012-10-05

**Authors:** Claudia Colomba, Raffaella Rubino, Lucia Siracusa, Francesco Lalicata, Marcello Trizzino, Lucina Titone, Manlio Tolomeo

**Affiliations:** 1 Dipartimento di Scienze per la promozione della salute – Sezione di Malattie infettive, Università di Palermo, Via del Vespro, 129-90127 , Palermo, Italy

**Keywords:** Tuberculosis, Myelofibrosis, Ruxolitinib

## Abstract

**Background:**

Primary myelofibrosis is a myeloproliferative disorder characterized by bone marrow fibrosis, abnormal cytokine expression, splenomegaly and anemia. The activation of JAK2 and the increased levels of circulating proinflammatory cytokines seem to play an important role in the pathogenesis of myelofibrosis. Novel therapeutic agents targeting JAKs have been developed for the treatment of myeloproliferative disorders. Ruxolitinib (INCB018424) is the most recent among them.

**Case presentation:**

To our knowledge, there is no evidence from clinical trials of an increased risk of tuberculosis during treatment with JAK inhibitors. Here we describe the first case of tuberculosis in a patient treated with Ruxolitinib, a male with a 12-year history of chronic idiopathic myelofibrosis admitted to our Institute because of fever, night sweats, weight loss and an enlarging mass in the left inguinal area for two months.

**Conclusion:**

Treatment with Ruxolitinib may have triggered the reactivation of latent tuberculosis because of an inhibition of Th1 response. Our case highlights the importance of an accurate screening for latent tuberculosis before starting an anti-JAK 2 treatment.

## Introduction

Primary myelofibrosis is a myeloproliferative disorder characterized by bone marrow fibrosis, abnormal cytokine expression, splenomegaly and anemia. The molecular mechanisms underlying pathogenesis are poorly understood. Recent studies have implicated mutations that directly or indirectly lead to a deregulated activation of tyrosine-protein kinases, Janus-activated kinase 2 (JAK2) [1,2]. Therefore, the activation of JAK2 and the increased levels of circulating proinflammatory cytokines seem to play an important role in the pathogenesis of myelofibrosis [3]. Novel therapeutic agents targeting JAKs have been developed for the treatment of myeloproliferative disorders. Ruxolitinib (INCB018424) is the most recent among them [4,5]. The suggested mechanism of action of Ruxolitinib is the attenuation of cytokine signaling via the inhibition of JAK1 and JAK2, resulting in antiproliferative and proapoptotic effects.

The earliest studies showed that Ruxolitinib provides reductions in splenomegaly and constitutional symptoms [6].

To our knowledge, there is no evidence from clinical trials of an increased risk of tuberculosis during treatment with JAK inhibitors [6]. Here we describe the first case of tuberculosis in a patient treated with Ruxolitinib.

## Case presentation

A male with a 12-year history of chronic idiopathic myelofibrosis was admitted to the Institute of Infectious Diseases, “Paolo Giaccone” University Polyclinic in Palermo, because of fever, night sweats, weight loss and an enlarging mass in the left inguinal area for two months. Our patient had been enrolled in the COMFORT-II study, a randomized, open-label Phase III study of oral JAK2 inhibitor Ruxolitinib versus best available therapy in patients with primary myelofibrosis, post-polycythemia vera myelofibrosis, and post-essential thrombocythemia myelofibrosis. On admission, physical examination showed a lymph node enlargment in the left inguinal area, crepitations at right lung base and diminished vesicular murmurs at left lung base on pulmonary auscultation, firm hepatosplenomegaly (DL 22.5 cm), peripheral edema, slow speech without signs of meningeal irritation. Laboratory test results revealed RBC 2770000 cells/mm^3^, Hb 8.1 g/dL, WBC 5490 cells/mm^3^ (N 64.8% L 26% M 8.4%), PLT 69000 cells/mm^3^, total and direct bilirubin 3.56/2.49 mg/dL respectively, albumin 2.7 g/dL, erythrocyte sedimentation rate (ESR) 15 mm, C-reactive protein (CRP) 6.94 mg/dL. The patient underwent inguinal lymphadenectomy and microbiological examination showed acid-alcohol resistant bacilli and positive polymerase chain reaction (PCR) for Mycobacterium tuberculosis. The chest radiograph revealed consolidation in the left middle lung field. A QuantiFERON-TB gold was performed with positive result (11.3 U/mL). M. tuberculosis was cultured from three sputum samples. Abdominal CT-scan showed confluent and colliquative para-aortic, inter aorta-cava, iliac and left inguinal lymph nodes. The standard tuberculosis treatment with isoniazid, rifampicin, pyrazinamide and ethambutol was started.

## Discussion

The main side effect of inhibitors of JAK1 and 2 can be an increased risk of infections, related to a depressed Th1 response and a reduced production of gamma interferon (INF-γ) [7].

IFN-γ is a key cytokine involved in protective immunity against Mycobacterium tuberculosis, regulating the expression of genes involved in antimycobacterial effector functions.

Mycobacterium tuberculosis leads to the activation of alveolar macrophages, with production of cytokines that limit the growth of ingested organisms. Alveolar macrophages and dendritic cells produce IL-12 and additional cyto- and chemokines such as TNF-α, IL-1, IL-6, IL-15, IL-18. IL-12 plays as a master regulator of Th1 response inducing the production of IFN-γ. IL-12 binds to a high affinity receptor (IL-12R) and activates Janus family tyrosine kinases, leading to phosphorylation of tyrosine residues of STAT3 and STAT4. The final event is the transcription of IFN-γ mRNA. IFN-γ activated macrophages produce bactericidal superoxide and reactive nitrogen intermediates, as well as IL-12, IL-1 and IL-6 [8].

## Conclusion

Treatment with Ruxolitinib may have triggered the reactivation of latent tuberculosis because of an inhibition of Th1 response. Our case highlights the importance of an accurate screening for latent tuberculosis before starting an anti-JAK 2 treatment.

## Consent

Written informed consent was obtained from the patient for publication of this case report. A copy of the written consent is available for review by the Editor-in-Chief of this journal.

## Competing interests

The authors declare that they have no competing interests*.*

## Authors’ contributions

CC, RR and LT analyzed clinical and therapeutic aspects of the case. LS, FL and MT conceived of the study, and participated in its design and coordination and helped to draft the manuscript. MT designed and participated as haematology specialist. All authors read and approved the final manuscript.
